# Type II alveolar epithelial cell aryl hydrocarbon receptor protects against allergic airway inflammation through controlling cell autophagy

**DOI:** 10.3389/fimmu.2022.964575

**Published:** 2022-07-22

**Authors:** Ji Wang, Yilin Zhao, Xin Zhang, Wei Tu, Rongjun Wan, Yingchun Shen, Yan Zhang, Ruchik Trivedi, Peisong Gao

**Affiliations:** ^1^ Division of Allergy and Clinical Immunology, Johns Hopkins School of Medicine, Baltimore, MD, United States; ^2^ Department of Respiratory and Critical Care Medicine, West China Hospital, Sichuan University, Chengdu, China; ^3^ Laboratory of Pulmonary Immunology and Inflammation, Frontiers Science Center for Disease-related Molecular Network, Sichuan University, Chengdu, China; ^4^ Department of Respiratory Medicine, Xijing Hospital, The Fourth Military Medical University, Xi’an, China; ^5^ Department of Integrated Traditional Chinese and Western Medicine, West China Hospital, Sichuan University, Chengdu, China; ^6^ Department of Respirology and Allergy, Third Affiliated Hospital of Shenzhen University, Shenzhen, China; ^7^ Department of Respiratory Medicine, Xiangya Hospital, Central South University, Changsha, China

**Keywords:** asthma, cockroach allergen, aryl hydrocarbon receptor, autophagy, AT2 cells

## Abstract

**Rationale:**

Aryl hydrocarbon receptor (AhR), a ligand-activated transcription factor, has been considered as an important regulator for immune diseases. We have previously shown that AhR protects against allergic airway inflammation. The underlying mechanism, however, remains undetermined.

**Objectives:**

We sought to determine whether AhR specifically in type II alveolar epithelial cells (AT2) modulates allergic airway inflammation and its underlying mechanisms.

**Methods:**

The role of AhR in AT2 cells in airway inflammation was investigated in a mouse model of asthma with AhR conditional knockout mice in AT2 cells (*Sftpc-Cre;AhR^f/f^*). The effect of AhR on allergen-induced autophagy was examined by both *in vivo* and *in vitro* analyses. The involvement of autophagy in airway inflammation was analyzed by using autophagy inhibitor chloroquine. The AhR-regulated gene profiling in AT2 cells was also investigated by RNA sequencing (RNA-seq) analysis.

**Results:**

*Sftpc-Cre;AhR^f/f^* mice showed exacerbation of allergen-induced airway hyperresponsiveness and airway inflammation with elevated Th2 cytokines in bronchoalveolar lavage fluid (BALF). Notably, an increased allergen-induced autophagy was observed in the lung tissues of *Sftpc-Cre;AhR^f/f^* mice when compared with wild-type mice. Further analyses suggested a functional axis of AhR-TGF-β1 that is critical in driving allergic airway inflammation through regulating allergen-induced cellular autophagy. Furthermore, inhibition of autophagy with autophagy inhibitor chloroquine significantly suppressed cockroach allergen–induced airway inflammation, Th2 cytokines in BALFs, and expression of autophagy-related genes LC3 and Atg5 in the lung tissues. In addition, RNA-seq analysis suggests that autophagy is one of the major pathways and that *CALCOCO2/NDP52* and *S1009* are major autophagy-associated genes in AT2 cells that may contribute to the AhR-mediated cockroach allergen–induced airway inflammation and, subsequently, allergic asthma.

**Conclusion:**

These results suggest that AhR in AT2 cells functions as a protective mechanism against allergic airway inflammation through controlling cell autophagy.

## Introduction

Asthma is a leading serious chronic illness of children and adults worldwide, and its prevalence has reached unprecedented levels over the past few decades (1). Environmental allergen exposure has been considered to be one of the major risk factors for asthma (2). Of these, cockroach allergen is one of the major sources of indoor allergens and can give rise to Immunoglobulin E (IgE) sensitization and, subsequently, development of asthma (3–5). Furthermore, a recent study suggested that serum IgE levels specific to cockroach allergen were correlated with Th2 polarization (6). However, it remains unclear about the biological mechanisms underlying the cockroach allergen exposure-induced Th2-associated airway inflammation in asthma. Airway epithelium is the first line of defense against inhaled allergens. Interaction of airway epithelium with allergens leads to the release of inflammatory mediators, resulting in inappropriate recruitment of immune cells and skewing of downstream immune responses. However, the mechanisms underlying the allergen-induced mediator release from epithelial cells are still not completely understood.

The aryl hydrocarbon receptor (AhR) is a ligand-activated transcription factor widely expressed in barrier organs, such as airway, gut, and skin (7, 8), and in different cell types [e.g., DCs, Th17 and Treg cells, ILCs, and mesenchymal stem cells (MSCs)] (9–12). AhR is originally characterized for its function in the metabolism of environmental toxicants such as dioxins and many other polycyclic aromatic hydrocarbons (PAHs) (13–16). Recent studies have suggested a central role for AhR as an environmental sensing molecule in cell growth, cell differentiation, cell immune responses, and respiratory function (17–21). Not surprisingly, AhR has emerged as an attractive therapeutic target for different diseases including asthma (14–16, 22). Indeed, AhR has been associated with environmental pollutant–induced allergic airway inflammation (18, 23–25) and reactive oxygen species (ROS)-dependent mast cell degranulation and activation (26, 27). Our previous findings have suggested that benzo(a)pyrene (BaP, a ubiquitous air pollutant) co-exposure with Der f 1 (a common allergen to human) exacerbated AhR signaling pathway that regulates the co-exposure–induced airway epithelial cell oxidative stress and cytokine release (28, 29). Furthermore, our recent study demonstrated that epithelial AhR is essential in protecting against cockroach allergen–induced ROS generation, NLRP3 inflammasome activation, and Muc5ac expression (30). However, the specific role of AhR signaling in regulating allergic airway inflammation and its precise mechanisms has not been fully elucidated.

Autophagy is an the endogenous housekeeping process in maintaining cell homeostatic process by delivering damaged proteins and redundant cellular organelles to lysosomes for degradation during cellular stress (2). Evidence shows that autophagy is critical in shaping cellular immune responses and progression of inflammatory diseases. It was found that autophagy was increased in peripheral blood cells and sputum granulocytes from severe asthmatic patients (31). The increased autophagy has also been associated with asthma pathological processes such as extracellular matrix deposition, airway remodeling, and immune dysregulation (32–35). Previously, we found that inhibition of autophagy led to attenuated cockroach allergen–induced airway epithelial ROS generation, cytokine release, and subsequent allergic airway inflammation, suggesting a role for autophagy in promoting asthma development (36). However, it remains unclear how cellular autophagy is regulated in the pathogenesis of asthma.

In the present study, we focused on type II alveolar epithelial cells (AT2) to determine whether AhR, specifically in AT2 cells, is involved in regulating cellular autophagy and, subsequently, allergic airway inflammation. In particular, we generated AhR conditional knockout mice in AT2 cells (*Sftpc-Cre;AhR^f/f^*) and investigated the role of AhR specifically in AT2 cells in cockroach allergen–induced airway inflammation. We then uncovered a unique role for AhR in regulating allergen-induced cellular autophagy by both *in vivo* and *in vitro* analyses. We further elucidated an overexpression of autophagy in the lung tissues of asthma mouse model and inhibition of autophagy suppressed allergic airway inflammation. Most importantly, RNA sequencing (RNA-seq) analysis identified autophagy to be one of the most AhR-regulated biological processes in AT2 cells. Further analyses suggest that *CALCOCO2* and *S100a9* are the major AhR-regulated autophagy-associated genes. Overall, this study provides evidence that AhR regulates allergen-induced autophagy that may contribute to the protective mechanism of AhR signaling against allergic airway inflammation.

## Methods

### Mice

*Sftpc-Cre;AhR^f/f^* mice on the C57BL/6 background were generated by cross-breeding *Sftpc-cre* with *AhR^f/f^* mice in this study. *Sftpc-cre* mice were provided by Michael A. O’Reilly at the University of Rochester Medical Center (Rochester, NY, USA), and *AhR^f/f^* mice were purchased from the Jackson Laboratory (Bar Harbor, ME, USA). All mice were maintained under specific pathogen–free conditions at the animal facility of the Johns Hopkins University School of Medicine. The animal care and experiments were performed in compliance with the institutional and US National Institutes of Health guidelines and were reviewed and approved by the Johns Hopkins University Animal Care and Use Committee. All mice were used at 6–8 weeks of age, and all experiments used age- and sex-matched controls.

### Cockroach allergen–induced asthma mouse model

**Figure 1:**
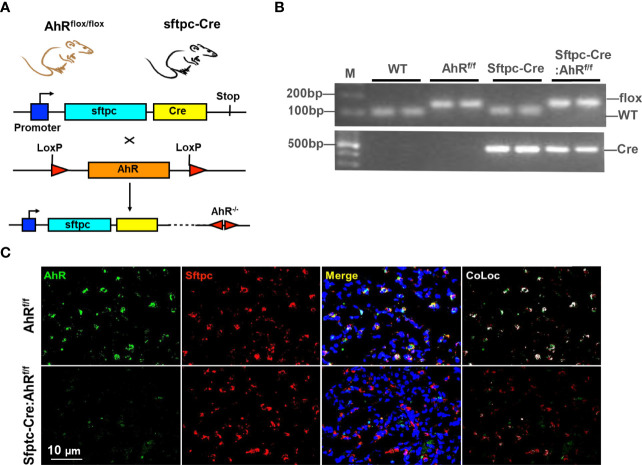
Generation of type II alveolar epithelial cell–specific AhR knockout mice. **(A)** Schematic representation of the crossbreeding of a floxed AhR mouse (*AhR^f/f^*) with a Sftpc-cre mouse. **(B)** Confirmation of *Sftpc-cre: AhR^f/f^* mice by genotyping. **(C)** AhR deletion in AT2 cells of *Sftpc-cre: AhR^f/f^* mice was confirmed by co-immunostaining with Sftpc and AhR in the lung tissues.

The cockroach allergen–induced asthma mouse model was established with the protocol as illustrated in **Figure 1A**. Briefly, mice were sensitized by intra-tracheal (i.t.) inhalation of 20 µg of cockroach extract (CRE, B46, GREER Laboratories) per mouse in 50 μl of Phosphate-Buffered Saline (PBS) under light anesthesia with isoflurane and challenged with the same amount of CRE. Control mice were treated with PBS. Mice were sacrificed, and samples [lung tissues, bronchoalveolar lavage fluid (BALF), and blood] were collected on the next day after the last allergen challenge (30). In particular, BALF was harvested by two consecutive flushes of the lung with 1.0 ml of ice-cold PBS. Blood was taken to screen for serum antibodies against cockroach allergen. In some cases, mice were pre-treated with autophagy inhibitor chloroquine (CLQ; C6628, 60 mg/kg, Sigma-Aldrich, St. Louis, MO, USA) or saline vehicle control by intraperitoneal administration 1 h before every single allergen challenge.

### Airway hyperresponsiveness

Mice were anesthetized with a ketamine (90 mg/kg)/xylazine (18 mg/kg) mixture, and a tracheotomy tube was inserted. Ventilation was initiated with a volume-cycled ventilator (Flexivent; SCIREQ Scientific) with a positive-end expiratory pressure of 2 cmH2O). Airway responsiveness was monitored by challenging mice with a dose-dependent aerosolized methacholine (0–30 mg/ml). The airway resistance was measured with the Flexivent software and exported to Pulmodyn data-acquisition software (Hugo Sachs Electronic) for data analysis (37).

### Histological analysis

Mouse lungs were perfused with 10 ml of ice-cold PBS injected into the right ventricle followed by excision, fixed with 4% formalin, and embedded in paraffin. Sections (4 µm) were then prepared from these paraffin-embedded lungs and subjected to hematoxylin and eosin (H&E) and periodic acid–Schiff (PAS) staining to evaluate general morphology and mucus production as previously described (30).

### Flow cytometry analysis

Single-cell suspensions were prepared from BALFs, and total cells in BALFs were counted by Countless II (ThermoFisher). Cellular differential percentages in BALFs were measured by flow cytometry on an Accuri C6 Plus Flow Cytometer (BD Biosystems), and the data were analyzed with FlowJo software (Tree Star Inc.) as previously described (38).

### Immunofluorescence staining

Immunofluorescence staining was performed as previously reported (37). Briefly, sectioned lung tissues were first blocked using 5% w/v BSA at room temperature for 1 h, followed by incubation with the primary antibodies as listed in the online repository (**Supplementary Table 1**) overnight at 4°C. The sample sections were then incubated with secondary antibodies conjugated with fluorescence at room temperature for 1 h. Isotype-matched negative control antibodies were used under the same conditions. The sections were mounted with the Fluoromount Mounting medium (Sigma) with DAPI (4′,6-diamidino-2-phenylindole) (ThermoFisher) and then observed by a NIKON ECLIPSE Ti-U microscope equipped with DS-Fi2 camera (NIKON, USA).

### Enzyme-linked immunosorbent assay

Interleukin-4 (IL-4), IL-5, Interferon-gamma (IFN-γ), IL-17A, IL-25, and Transforming growth factor-beta 1 (TGF-β1) in cell-free BALF or supernatant were quantified by using the Ready-Set-Go! ELISA (ThermoFisher) according to the manufacturer’s instructions. Serum levels of cockroach allergen–specific IgE and IgG1 were analyzed by Enzyme-linked immunosorbent assay (ELISA) as previously described (36).

### Cell culture and treatment

Human bronchial epithelial cells (HBEC3-KT, ATCC CRL-4051) were cultured in Ham’s F-12K (Kaighn’s) medium supplemented with 10% v/v FBS and 1% penicillin‐streptomycin. The cells were maintained at 37°C in a humidified atmosphere at 5% CO_2_. HBECs were treated with cockroach extract (CRE, Greer Laboratory) alone or pre-treated with AhR agonist TCDD (Sigma, 45899; 1.0 µM) or antagonist CH-223191 (Sigma, C8124; 10 µM) or vehicle controls 1 h before CRE treatment.

### Isolation of mouse alveolar type 2 cells

Mouse AT2 cells were isolated from *AhR^fl/fl^* mice and *Sftpc-Cre;AhR^flox/flox^* mice as previously reported (38). Briefly, mice were euthanized with ketamine and xylazine intraperitoneally. Lung tissues from these mice were digested with Dispase (50 units/ml, Gibco) at room temperature for 45 min and then incubated with AT2 isolation medium consisting of a 1:1 mixture of Dulbecco’s modified Eagle’s medium (DMEM) and Ham’s F-12 (DMEM/F-12; Sigma) supplemented with 0.01% DNase I (Sigma), sodium penicillin G (100 U/ml), and streptomycin (100 µg/ml). The mixed cells were filtered through cell strainers; stained with biotinylated anti-CD16/32, anti-CD45, anti-Ter119, and anti-Sca-1 (BioLegend); and incubated at 37°C shaking incubator for 30 min. These cells were further negatively selected with streptavidin-conjugated magnetic beads for 30 min at room temperature. Cells were centrifuged for 10 min at 300*g* at 4°C and incubated on dishes pre-coated with mouse IgG. After incubation for 2 h, non-adherent cells were collected, centrifuged, and re-suspended with AT2 isolation medium.

### RNA-seq analysis

RNA-seq libraries were prepared for sequencing using an Illumina TruSeq stranded mRNA sample preparation kit following the manufacturer’s recommended procedure. Briefly, total RNA was extracted using the RNeasy Mini Kit (QIAGEN). mRNA was enriched using oligo dT beads and then fragmented chemically by incubating at 94°C for 8 min. cDNA was synthesized with SuperScript II. After purification using AMPure XP beads, the double-stranded cDNA was ligated to TruSeq RNA adapters followed by 15 cycles of amplification and library purification. Sequencing was performed on an Illumina NextSeq6000. RNA-seq reads were aligned to the mouse reference genome GRCm38 using STAR aligner version 2.7.2b. BAM file outputs from STAR were annotated using Partek Genomic Suite (v6.6) and the RefSeq database. The gene expression levels were defined as counts by Salmon (39) and then normalized by log_2_-transformed counts per million (CPM, edgeR version 3.34.1) (40). Different expression analyses were performed with multiple linear regression models and empirical Bayes moderation embedded in the limma-trend approach (limma version 3.48.3) (40). Genes with absolute log_2_ fold change [abs(log_2_FC)] > 0.5 and *p* < 0.05 were defined as differentially expressed genes (DEGs). Gene set enrichment analyses was performed by the clusterProfiler 4.0 version 4.0.5 (41). Up- or downregulated DEGs were visualized by ComplexHeatmap (version 2.8.0) and ggplot2 (version 3.3.5) (42). All RNA-seq–based downstream analyses and visualizations were performed in R version 4.1.2. (see RNA-seq data used for analysis in **Supplementary Materials**).

### Transfection of siRNA

AhR knockdown in HBECs was accomplished by using a pre-designed MISSION siRNA small interfering RNA pair (Sigma-Aldrich, SASI_Hs01_00140198). siRNA transfection was performed with Lipofectamine™ RNAiMAX (ThermoFisher) following the manufacturer’s instruction, whereas control cells received negative control siRNA. Cells were cultured for 48 h, and transfection efficiency was evaluated by quantitative real-time PCR and Western blot.

### RNA isolation and quantitative real-time PCR analysis

Total RNA from lung tissues or HBECs was isolated using the Monarch Total RNA Miniprep Kit (New England Biolabs), and cDNAs were synthesized with the High-Capacity cDNA Reverse Transcription Kit (ThermoFisher). Quantitative real-time PCR analysis was performed using the Power SYBR Green PCR Master Mix (ThermoFisher) on an ABI Prism 7300 detection system. Data were analyzed using the 2^−ΔΔCT^ method relative to the housekeeping gene Actin (43). Primer sequences are provided in the Online Repository (see **Table E1**).

### Western blotting

Tissues or cells were lysed with an radioimmunoprecipitation assay (RIPA) buffer containing protease and phosphatase inhibitor cocktails (Sigma-Aldrich). Protein concentrations were measured by using a BCA kit (Thermo Fisher). An equal amount of total protein (20–50 µg) was loaded onto a 4%–12% Tris-Glycine Gel in NuPAGE MOPS SDS Running Buffer (Thermo Fisher Scientific) and then transferred using the iBlot2 NC Stack System (ThermoFisher). The membranes were blocked in 5% non-fat milk in Tris buffered saline with Tween® 20 (TBST) for 1 h at room temperature and then probed with primary antibodies overnight at 4°C. Species-appropriate secondary antibodies conjugated to IRDye 680RD or IRdye 800CW (LI-COR Biosciences) were used according to the manufacturer’s instructions. Detection was performed using iBright 1500 Imaging Systems, and fluorescent intensity was quantified using the system built in IBright Analysis Software (Thermo Fisher Scientific).

### Statistical analysis

All statistical analyses were carried out using GraphPad Prism version 8.2.1 (GraphPad Software, La Jolla, CA, USA). All data are presented as means ± SEM. Comparison of two groups was performed by student’s two-tailed unpaired t-test. Comparison of more than two groups was performed by ordinary one-way ANOVA followed by Tukey’s *post hoc* test. *P* < 0.05 was considered statistically significant.

## Results

### Generation of type II alveolar epithelial cell–specific AhR knockout mice

Airway epithelial cells are the first line of defense against inhaled particulate antigens, and epithelial activation is one of the characteristics of asthma (44). We have found that expression of AhR was significantly increased in the airways of asthmatic patients (45) and the mouse model of asthma (12). To determine the significance of epithelial AhR in cockroach allergen–induced asthma, we deleted AhR selectively from type II alveolar airway epithelium (AT2) by crossing floxed AhR mice (*AhR^f/f^*) with *Sftpc-cre* mice that express the Cre recombinase in AT2 cells from the endogenous promoter/enhancer elements of the surfactant protein C (Sftpc) locus (*Sftpc-Cre; AhR^f/f^*, **Figure 1A**). The mice were confirmed by genotyping (**Figure 1B**). The deletion of AhR in AT2 cells was further confirmed by co-immunostaining with AhR and Sftpc in the lung tissues (**Figure 1C**). Thus, these mice represent an ideal mouse model to examine the functional role of AhR in AT2 cells in allergic airway inflammation.

### Type II alveolar epithelial cell AhR protects against allergic airway inflammation

**Figure 2:**
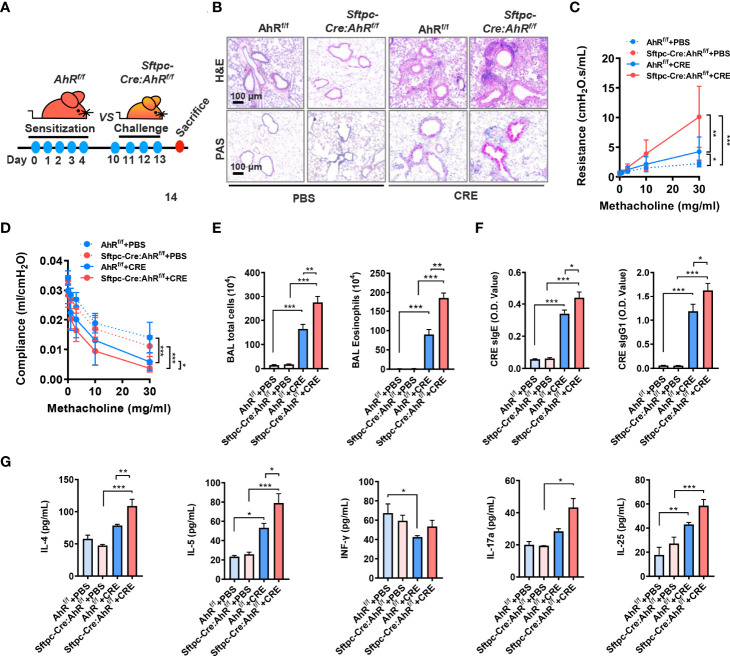
Type II alveolar epithelial cell AhR protects against airway hyperresponsiveness and allergic airway inflammation. **(A)** Experimental scheme for cockroach allergen–induced mouse model of asthma. **(B)** Histological examination of mouse paraffin lung sections stained with hematoxylin and eosin (H&E, upper panel) and Alcian Blue periodic acid–Schiff (AB-PAS, lower panel). **(C, D)** Lung resistance **(C)** and compliance **(D)** in response to increasing concentrations of methacholine using the forced oscillation technique (FlexiVent, SCIREQ). **(E)** Bronchoalveolar lavage (BAL) fluid total and eosinophil cell counts as assessed by flow cytometry. **(F)** Serum levels of cockroach allergen–specific IgE and IgG1. **(G)** Levels of cytokines in BALFs. n = 8–10. Data represent means ± SEM. *p < 0.05, **p < 0.01, and ***p < 0.001.

Next, we used these newly generated *Sftpc-Cre;AhR^f/f^* mice to create an asthma mouse model by using our previous protocol as illustrated in **Figure 2A**. Histological analysis demonstrated that CRE-treated *Sftpc-Cre;AhR^f/f^* mice had an increased recruitment of inflammatory cells to the lung with dense peribronchial infiltrates (**Figure 2B**, upper panel) and goblet cell hyperplasia (**Figure 2B**, lower panel) as compared with *AhR^f/f^* mice. Consistently, these *Sftpc-Cre;AhR^f/f^* mice showed a significant increase in airway resistance (**Figure 2C**) and decrease in airway compliance (**Figure 2D**). Compared with *AhR^f/f^* mice, the total inflammatory cells, especially eosinophils, were remarkably increased in the BALFs of *Sftpc-Cre;AhR^f/f^* mice (**Figure 2E**). Higher levels of cockroach allergen–specific IgE and IgG1 were observed in serum of CRE-treated *Sftpc-Cre;AhR^f/f^* (**Figure 2F**). Furthermore, *Sftpc-Cre;AhR^f/f^* mice had higher levels of IL-4 and IL-5 in BALFs (**Figure 2G**). In contrast, no clear change was observed for IFN-γ, IL-17, and IL-25. Collectively, these findings indicate that AhR in AT2 cells protects against cockroach allergen–induced airway hyperresponsiveness and Th2-associated allergic airway inflammation.

### Type II alveolar epithelial cell AhR protects against allergen-induced autophagy in a mouse model of asthma

**Figure 3:**
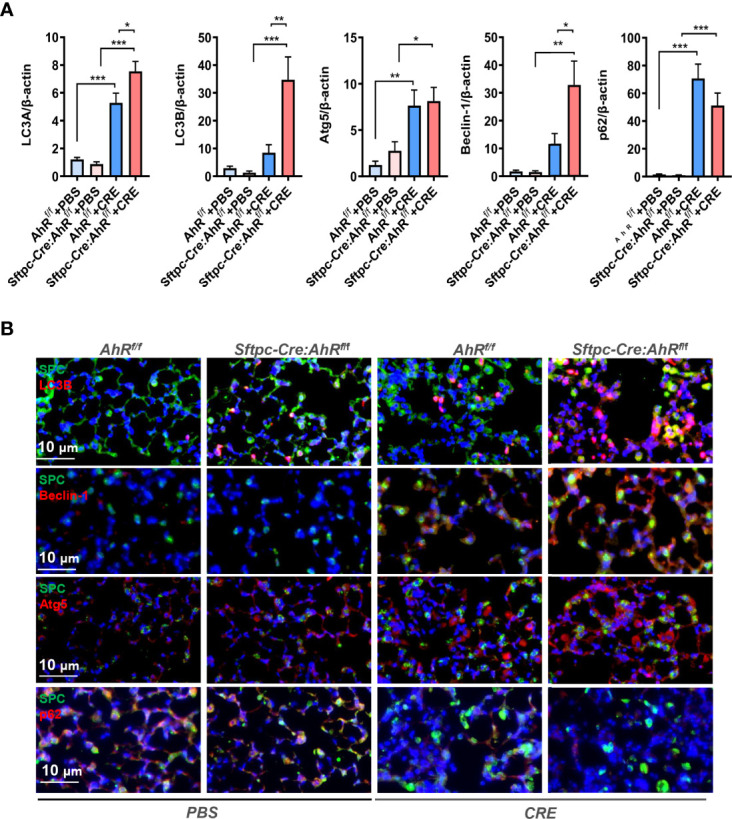
Type II alveolar epithelial cell AhR protects against allergen-induced autophagy in a mouse model of asthma. **(A)** RT-PCR analyses of autophagy-related genes in the lung tissues of asthma mouse model. n = 8–12. **(B)** Representative images of co-immunofluorescence staining of autophagy-related genes with AT2 marker SPC. n = 6. Data in **(A)** represent means ± SEM. *p < 0.05, **p < 0.01, and ***p < 0.001.

Previously, we have demonstrated that excessive autophagy is critical in cockroach allergen–induced ROS generation, cytokine release, and subsequent allergic airway inflammation (36). To determine whether epithelial AhR regulates cockroach allergen–induced autophagy, we detected expression of autophagy-related genes in the lung tissues of CRE-induced mouse model with *AhR^f/f^* or *Sftpc-Cre;AhR^f/f^* mice. Compared with PBS-treated mice, increased expression of LC3A, Atg5, and p62 was seen in the lung tissues of CRE-sensitized and challenged mice as analyzed by RT-PCR (**Figure 3A**). Interestingly, compared with *AhR^f/f^* mice, the increased expression of ILC3A, LC3B, and Beclin-1 was further enhanced in CRE-treated *Sftpc-Cre;AhR^f/f^* mice. The increased autophagy was specifically confirmed in AT2 cells by co-immunofluorescent staining. The CRE-treated *Sftpc-Cre;AhR^f/f^* mice showed a significantly increased expression of LC3B in AT2 cells compared with *AhR^f/f^* mice (**Figure 3B**). Similar patterns were noted for Beclin-1 and ATG5, indicating that AhR in AT2 cells protects against cockroach allergen–induced autophagy.

### Aryl hydrocarbon receptor modulates allergen-induced autophagy in human bronchial epithelial cells

**Figure 4:**
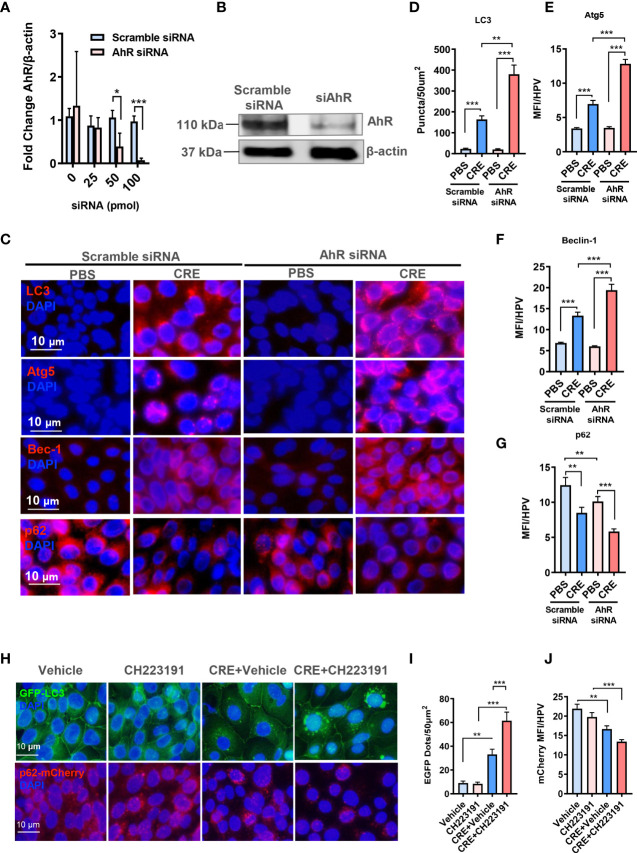
Aryl hydrocarbon receptor modulates allergen-induced autophagy in human bronchial epithelial cells (HBECs). **(A)** RT-PCR confirmation of AhR knockdown in HBECs by using different doses of AhR siRNA. **(B)** AhR knockdown in HBECs with 100-pmol AhR siRNA was further confirmed by Western blot. **(C)** Representative images of immunofluorescence staining of autophagy-related genes in HBECs with or without AhR knockdown. n = 6. **(D–G)** Quantification of immunofluorescence staining for autophagy-related genes LC3 **(D)**, Atg5 **(E)**, Beclin-1 **(F)**, and p62 **(G)** in HBECs with or without AhR knockdown. n = 12. **(H)** Representative immunofluorescence images of CRE-treated HBECs expressing GFP-LC3 or p62-mCherry in the presence or absence of AhR antagonist CH223191. **(I)** Quantitative analysis of autophagic flux according to the number of LC3 puncta (GFP dots/50 µm^2^). **(J)** Quantitative analysis of p62-mCherry florescent signal in **(H)**. n = 12 [four different high-power fields (hpfs) of three independent experiments]. Data represent means ± SEM. *p < 0.05, **p < 0.01, and ***p < 0.001.

To further confirm the functional significance of AhR in modulating autophagy, we investigated whether AhR knockdown can affect cockroach allergen–induced autophagy by *in vitro* analysis. AhR was knocked down in HBECs by using different doses of siRNA, and siRNA at 100 pmol showed the best deletion of AhR as assessed by RT-PCR (**Figure 4A**). AhR knockdown was further confirmed by Western blot (**Figure 4B**). As expected, immunostaining analysis illustrated that CRE exposure induced autophagy in HBECs as assessed by the increased expression of LC3 (**Figures 4C, D**), ATG5 (**Figures 4C, E**), and Beclin-1 (**Figures 4C, F**) but decreased expression of p62 (**Figures 4C, G**). Of interest, the increased expression of LC3 (**Figures 4C, D**), ATG5 (**Figures 4C, E**), and Beclin-1 (**Figures 4C, F**) was further enhanced in HBECs with AhR knockdown. To visualize the regulation of autophagy by AhR, we transfected either a GFP-LC3 or p62-mCherry construct in HBECs and then treated them with AhR antagonist CH223191. As shown in **Figure 4H**, the autophagic flux induced by CRE was increased according to the number of LC3 puncta (**Figures 4H, I**). The increased autophagy was potentiated in HBECs pre-treated with AhR antagonist CH223191. In contrast, expression of p62 was significantly reduced in HBECs treated with CRE in relative to PBS. No statistical difference was observed for HBECs with or without CH223191 pre-treatment (**Figures 4H, J**). Taken together, our *in vitro* analysis supports that epithelial AhR regulates cockroach allergen–induced autophagy.

### Inhibition of autophagy attenuates allergic airway inflammation

**Figure 5:**
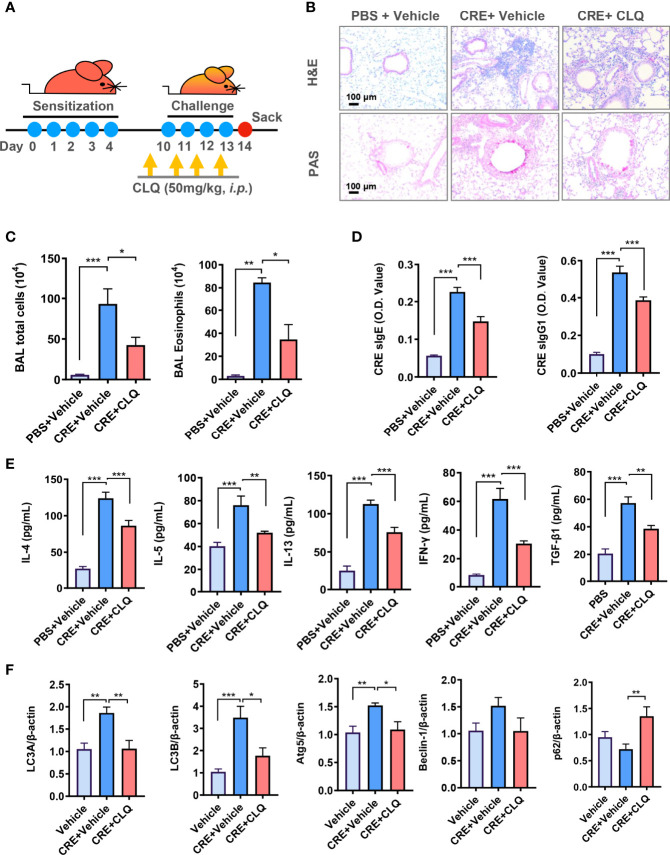
Inhibition of autophagy attenuates allergic airway inflammation. **(A)** Experimental scheme for cockroach allergen–induced mouse model of asthma in the presence or absence of autophagy inhibitor chloroquine (CLQ). **(B)** Histological examination of mouse paraffin lung sections stained with hematoxylin and eosin (H&E, upper panel) and periodic acid–Schiff (PAS, lower panel). **(C)** Bronchoalveolar lavage (BAL) fluid total and eosinophil cell counts as assessed by flow cytometry. **(D)** Serum levels of cockroach allergen–specific IgE and IgG1. **(E)** Levels of cytokines in BALFs. **(F)** RT-PCR analyses of autophagy-related genes in the lung tissues of asthma mouse model. n = 10. Data represent means ± SEM. *p < 0.05, **p < 0.01, and ***p < 0.001.

Given the significance of autophagy in AT2 cell AhR-modulated allergic airway inflammation, we asked whether inhibition of autophagy can suppress cockroach allergen–induced airway inflammation by using autophagy inhibitor CLQ in our mouse model following the protocol illustrated in (**Figure 5A**). CLQ is the most widely used classic inhibitor of autophagy that inhibits the last stage of autophagy (46). Consistent with our previous or current findings, CRE exposure induced airway inflammation when compare with PBS treatment. When these CRE-treated mice were pre-exposed to CLQ, the CRE-induced airway inflammation was significantly attenuated. In particular, CLQ pre-treatment inhibited CRE-induced peribronchial inflammation (H&E) (**Figure 5B**, upper panel) and goblet cell hyperplasia (PAS) (**Figure 5B**, lower panel). These CLQ-treated mice also showed reduced numbers of total inflammatory cells, particularly eosinophils in BALFs (**Figure 5C**). Moreover, cockroach allergen specific IgE and IgG1 in serum (sIgE and sIgG1) were reduced in mice treated with CLQ (**Figure 5D**). Furthermore, CLQ treatment led to the significant reduction in the levels of IL-4, IL-5, IL13, IFNγ, and TGF-β1 in BALFs (**Figure 5E**). Furthermore, as expected, treatment with CLQ attenuated CRE-induced expression of LC3A, LC3B, and Atg5 but led to the accumulation of p62 (**Figure 5F**). These results further support that inhibition of autophagy can prevent cockroach allergen–induced Th2-associated airway inflammation.

### TGF-β1 is required in AhR-mediated cockroach allergen–induced autophagy

**Figure 6:**
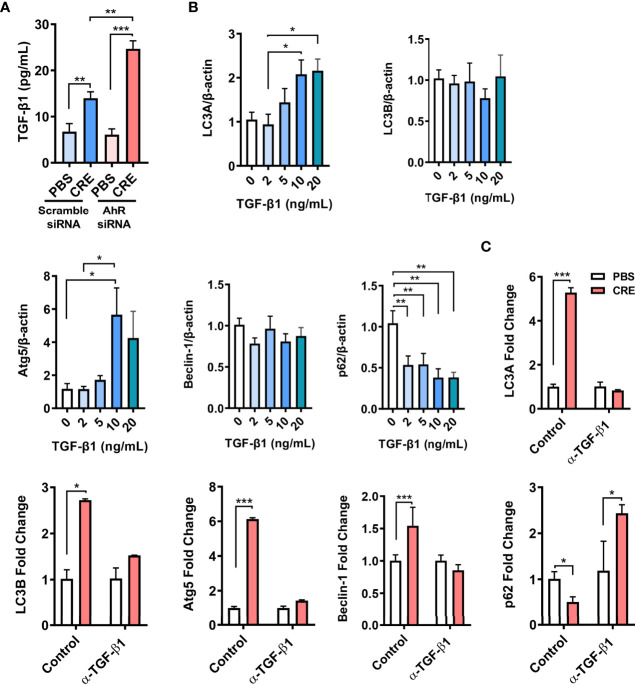
TGF-β1 is required in AhR-mediated cockroach allergen–induced autophagy. **(A)** ELISA analysis of TGF-β1 levels in supernatants of cockroach allergen (CRE)–treated HEBCs with or without AhR siRNA knockdown. **(B)** Expression of LC3A, LC3B, Atg5, Beclin-1, and p62 in the recombinant TGF-β1–treated HBECs at different concentrations. **(C)** Expression of LC3A, LC3B, Atg5, Beclin-1, and p62 in cockroach allergen (CRE-treated) HBECs in the presence or absence of TGF-β1 neutralizing antibody (α-TGF-β1). Data represent means ± SEM of at least two independent experiments. *p < 0.05, **p < 0.01, and ***p < 0.001.

We have previously reported that AhR modulates allergen-induced epithelial TGF-β1 secretion and signaling activation (29). We hypothesized that TGF-β1 may be a key player in AhR-mediated cockroach allergen–induced autophagy. AhR in HEBCs was knocked down by siRNA s illustrated in (**Figures 4A, B**). These HBECs were exposed to CRE for 24 h, and levels of TGF-β1 in supernatants were measured by ELISA. As noted, cockroach allergen exposure induced significant levels of TGF-β1 release from HBECs (**Figure 6A**). Of interest, the TGF-β1 release was enhanced in HEBCs with AhR knockdown. These findings provide further evidence that AhR modulates cockroach allergen–induced epithelial TGF-β1 secretion. To determine the role of TGF-β1 in autophagy, HBECs were treated with different doses of recombinant TGF-β1 for 24 h, the expression of autophagy-associated genes was detected by RT-PCR. Significantly increased LC3A and Atg5, but reduced p62 expression was observed in TGF-β1–treated HBECs (**Figure 6B**). No clear change was noted for Beclin-1. To further investigate whether TGF-β1 is required in cockroach allergen–induced autophagy, TGF-β1 neutralizing antibody (α-TGF-β1) was used to remove TGF-β1 in supernatants of CRE-treated HBECs. Intriguingly, the CRE-induced increased expression of LC3A, LC3B, Atg5, and Beclin-1 was almost completely attenuated in α-TGF-β1 pre-treated HBECs (**Figure 6C**). In contrast, α-TGF-β1 treatment induced increased expression of p62 that was reduced in CRE-treated HBECs. These findings support the hypothesis that TGF-β1 is essential in AhR-mediated cockroach allergen–induced autophagy.

### RNA-seq analysis identifies autophagy as one of the major AhR-regulated signaling pathways in AT2 cells

**Figure 7:**
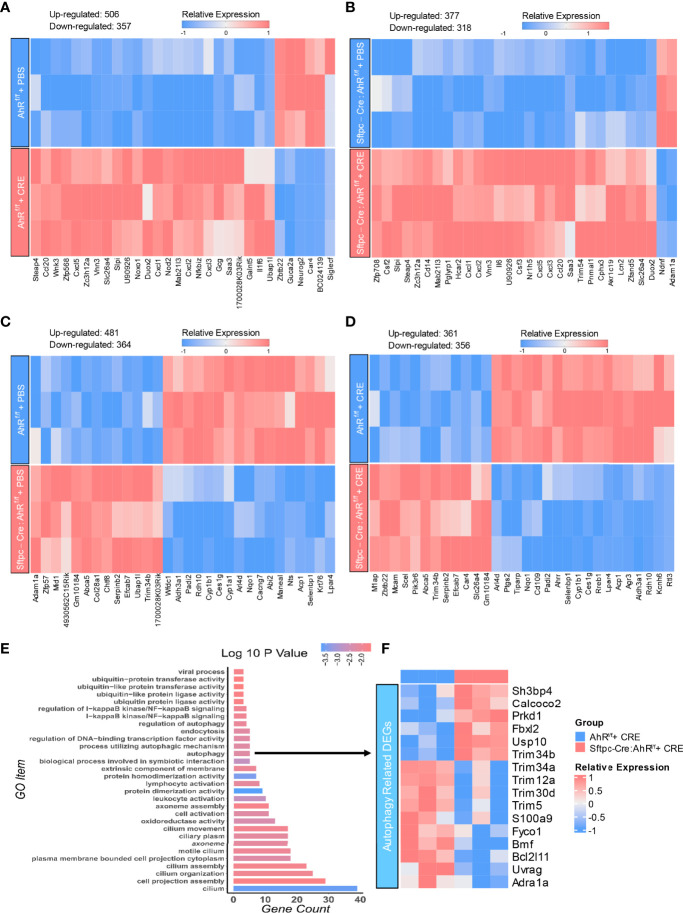
AhR-regulated transcriptional profiles in AT2 cells. **(A, B)** Heatmap of differentially expressed mRNAs in AT2 cells of *AhR^f/f^* **(A)** or *Sftpc-cre;AhR^f/f^* **(B)** mice treated with PBS verses cockroach allergen (CRE, 50 µg/ml) for 24 h **(C, D)** Heatmap of differentially expressed mRNAs in AT2 cells of *AhR^f/f^* verses *Sftpc-cre;AhR^f/f^* mice treated with PBS **(C)** or cockroach allergen CRE, 50 µg/ml) **(D)** for 24 h n = 3 per group **(A–D)**. **(E)** Gene Ontology (GO) functional enrichment analysis of the differentially expressed genes in **(D)**. **(F)** Heatmap of differentially expressed genes (DEG) that are involved in cellular autophagy.

To further examine the underlying mechanisms by which epithelial AhR modulates allergic airway inflammation, we analyzed the transcriptional profiles of AT2 cells isolated from the lungs of *AhR^f/f^* and *Sftpc-cre;AhR^f/f^* mice that were treated with CRE (50 µg/ml) for 24 h. The pattern of the up- and downregulated genes is illustrated by heatmap. First comparisons were made in AT2 cells treated with either PBS or CRE. Results revealed approximately 863 mRNAs that were differentially expressed in AT2 cells from *AhR^f/f^* mice, including 357 downregulated (e.g., *Siglec-F*) and 506 upregulated genes [*Ccl20*, *Cxcl2*, *NOXO1*, *Duox2*, and *Slc26a4* (38)] (**Figure 7A**). Approximately 685 mRNAs were differentially expressed in AT2 cells from *Sftpc-Cre;AhR^f/f^* mice, including 318 downregulated and 377 upregulated genes (**Figure 7B**). Next comparisons were made in AT2 cells between *AhR^f/f^* and *Sftpc-Cre;AhR^f/f^* mice. Results showed a total of 845 mRNAs differentially expressed in PBS-treated AT2 cells, including 364 downregulated (e.g., *Cyp1a1* and *Cyp1b1*) and 481 upregulated genes (**Figure 7C**). Approximately 717 mRNAs were differentially expressed in CRE-treated AT2 cells, including 356 downregulated [e.g., *Ahrr*, *Cyp1b1*, and *Ptgs2* (also called *COX-2*) (47)] and 361 upregulated genes [e.g., *Slc26a4* (38)] (**Figure 7D**). The DEGs in CRE-treated AT2 cells were further grouped into functional groups using the Gene Ontology (GO) functional enrichment analysis. Of interest, autophagy was identified to be one of the most enriched GO terms (**Figure 7E**). Several other significant GO terms were also identified, including ubiquitin-protein transferase activity, cell activation, and cilium-related terms (e.g., movement, assembly, organization, and cilium) (**Figure 7E**). Among all these autophagy-associated genes, *sh3bp4*, *Calcoco2*, *Prkd1*, *Fbxl2*, *Usp10*, and *Trim34b* were significantly upregulated, but *Trim34a*, *Trim12a*, *Trim30d*, *Trim5*, *S100a9*, and *BCL21ll* were downregulated (**Figure 7F**). These results support our previous findings that autophagy is one of the major AhR-regulated biological processes in AT2 cells.

### *CALCOCO2* and *S100a9* are the major AhR-regulated autophagy-associated genes in AT2 cells

**Figure 8:**
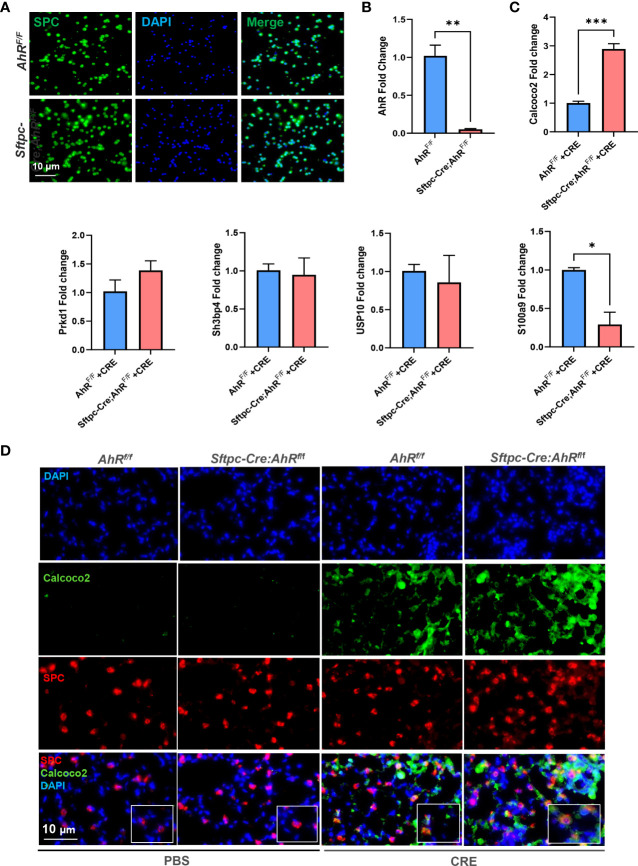
*Calcoco2* and *S100a9* are the major AhR-regulated autophagy-associated genes in AT2 cells. **(A)** Confirmation of AT2 cells isolated from *AhR^f/f^* or *Sftpc-Cre;AhR^f/f^* mice by immunostaining. **(B)** Confirmation of AhR deficiency in AT2 cells derived from *Sftpc-Cre;AhR^f/f^* mice by RT-PCR. **(C)** RT-PCR analysis of highly selected differentially expressed autophagy-associated genes of in AT2 cells. **(D)** Representative images of co-immunofluorescence staining of *Calcoco2* with AT2 marker SPC. Data represent means ± SEM of at least two independent experiments. *p < 0.05, **p < 0.01, and ***p < 0.001.

RT-PCR validation further narrowed down the gene list by focusing on several highly selected genes, including *Calcoco2*, *sh3bp4*, *Prkd1*, *Usp10*, and *S100a9.* AT2 cells were isolated from *AhR^fl/fl^* or *Sftpc-Cre;AhR^f/f^* mice and then confirmed by immunostaining (**Figure 8A**). AhR deficiency in AT2 cells derived from *Sftpc-Cre;AhR^f/f^* mice was further confirmed by RT-PCR (**Figure 8B**). Of these selected genes, *Calcoco2* showed a significant increase in CRE-treated AT2 cells from *Sftpc-cre;RhoA^f/f^* mice as compared with those from *AhR^f/f^* mice (**Figure 8C**). *Calcoco2* has been shown to initiate selective autophagy through recruiting Unc-51-like kinase (ULK) and TANK-binding kinase 1 (TBK1) kinase complexes (48, 49). Further analysis demonstrated an increased expression of *Calcoco2* in the lung tissues of our asthma mouse model as assessed by immunostaining (**Figure 8D**). The increased *Calcoco2* was further enhanced in *Sftpc-cre;RhoA^f/f^* mice as compared with control mice. Notably, the same pattern was observed for *Calcoco2* expression specifically in AT2 cells. In contrast to *Calcoco2*, RT-PCR analysis showed that *S100a9* expression was significantly reduced in AT2 cells from *Sftpc-cre;RhoA^f/f^* mice compared with those from *AhR^f/f^* mice (**Figure 8C**). S100a9 (S100 calcium binding protein A9) is a pro-inflammatory alarmin associated with inflammation-related diseases. Suppression of S100a9 has been implicated to protect against LPS-induced lung injury (50). Together, these data suggest a possible mechanism that AhR regulates cellular autophagy through modulating *Calcoco2/S100a9* expression.

## Discussion

AhR as a receptor for environmental contaminants has been shown to protect against allergic airway information and remodeling in allergic asthma by using global AhR knockout mice (18, 23–25, 51, 52). In the present study, we specially focused on AT2 cells by generating AhR knockout mice in AT2 cells (*Sftpc-Cre;AhR^f/f^*) and provided supporting evidence that AhR in AT2 cells prevents allergen-induced airway hyperresponsiveness and Th2-associated airway inflammation.

AT2 cells are recognized as progenitor cells in response to lung injury and have a unique function in providing pulmonary surfactant and a variety of inflammatory mediators needed for the special microenvironment in the alveolus (53, 54). AT2 cell damage has been implicated in lung fibrosis (55) and inflammation (56). However, the genetic regulation and functional significance of AT2 cells in allergic immune responses to environmental allergens in asthma remain unknown. Here, we generated the *Sftpc-Cre;AhR^f/f^* mice to determine whether AhR specifically in AT2 cells play an important role in allergic airway inflammation. *Sftpc-Cre;AhR^f/f^* mice with AhR deletion selectively from AT2 cells were generated by crossing *AhR^f/f^* with *Sftpc-cre* mice. We selected *Sftpc-cre* mice for AhR deletion in AT2 cells because Sftpc is highly expressed by AT2 cells and has been widely used as a genetic marker for AT2 cells (57–59). However, we recognize that Sftpc may not be uniquely expressed in AT2 cells (60–62), and future studies are thus needed to identify unique markers to further define the role of AT2 cells in allergic airway inflammation.

Using the newly generated *Sftpc-Cre;AhR^f/f^* mice, we found that AT2 cell–specific deletion of AhR led to an exacerbation of allergen-induced airway hyperresponsiveness and airway inflammation with elevated eosinophils and Th2 cytokines in BALFs. These results were consistent with our previously report with global AhR knockout mice. Especially, cockroach allergen–treated AhR^−/−^ mice showed exacerbation of airway inflammation when compared with WT mice, which was further supported by using an AhR agonist 2,3,7,8-tetrachlorodibenzo*-p-*dioxin (TCDD) (12). Furthermore, Chang et al. have also reported that AhR^−/−^ mice had exacerbated ovalbumin (OVA)–induced asthma symptoms, including airway inflammation, mucus production, and airway remodeling, and suggested that AhR is required in maintaining normal lung function and mediating disease severity (51). In contrast, Traboulsi et al. found that OVA-induced mouse model with AhR^−/−^ mice showed significantly enhanced eosinophilia and lymphocyte infiltrates into the airways with increased IL-4 and IL-5 in the airways, but no changes were observed for airway hyperresponsiveness (52). These discoveries highlight the complexity of AhR activation and the need for the investigation into how AhR specifically in AT2 cells regulates airway inflammation and airway hyperresponsiveness.

Our studies have suggested several underlying mechanisms as to how AhR regulates airway inflammation. We have provided evidence that AhR protects lungs from allergen-induced inflammation by modulating MSC recruitment and their immune-suppressive activity (12). Further studies suggest that active AhR signaling in MSCs regulates MSC suppressive activity by polarizing macrophages into anti-inflammatory M2 in asthma (63). Our very recent studies uncover a key functional axis of AhR-ROS-NLRP3 inflammasome that plays an important role in the development of allergic airway inflammation (30). In particular, we demonstrated that epithelial AhR plays a role in cockroach allergen–induced ROS generation and NLRP3 inflammasome activation that are essential in the development of allergic airway inflammation and mucus production. Intriguingly, MCC950, a NLRP3-specifc inhibitor, showed significant inhibition of cockroach allergen–induced airway inflammation and Muc5ac expression. In the current study, we focused on autophagy and demonstrated that AhR regulates allergen-induced cellular autophagy, and, subsequently, allergic airway inflammation. Autophagy is a homeostatic process in which eukaryotic cell encapsulates damaged proteins or organelles for lysosomal degradation and recycling (64). It has also been shown to shape cellular immune responses initiated by environmental pollutants, allergens, and respiratory tract infections (3, 34, 65–71). Abnormality in autophagy has been associated with several major asthma phenotypes, including airway hyperresponsiveness (66), inflammation (72), mucus hyperproduction (73), and remodeling (32). Accumulating evidence suggests that autophagy may be detrimental or beneficial in asthma that depends on the cell types involved (74). We have previously demonstrated that cockroach allergen can induce autophagy in human airway epithelial cells and excessive autophagy may contribute to cockroach allergen–induced airway epithelial ROS generation, cytokine release, and allergic airway inflammation (36). Importantly, autophagy has been shown to regulate NLRP3 inflammasome and secretion of proinflammatory cytokines (75), raising the possibility that autophagy may be a part of the AhR-regulated axis of ROS–inflammasome, which would be of interest to explore in the future.

In this study, we provide a critical but previously unrecognized mechanism that AhR may protect lung from allergic inflammation *via* regulating allergen-induced autophagy in AT2 cells. In particular, we found that *Sftpc-Cre;AhR^f/f^* mice showed an increased cockroach allergen–induced autophagy compared with *AhR^f/f^* mice. The increased autophagy was confirmed in AT2 cells of those mice. Although no human AT2 cells were available, the regulation of AhR in allergen-induced autophagy was further confirmed in HBECs with or without AhR knockdown, suggesting that the AhR regulation on AT2 cell autophagy might also be true for the bronchial airway epithelial cells. Importantly, this finding was further supported by direct monitoring of autophagic flux according to the number of GFP-LC3 puncta under fluorescence microscopy. CRE-treated HBECs showed an increased autophagic flux, which was further enhanced by the treatment with AhR antagonist CH223191. Most importantly, pre-treatment of mice with autophagy inhibitor CLQ abrogated cockroach allergen–induced airway inflammation, Th2-associated cytokines, and autophagy-related gene expression. CLQ is a classic inhibitor of autophagy that blocks the binding of autophagosomes to lysosomes (46). Collectively, these studies highlight a critical role for AhR in AT2 cells in preventing allergen-induced autophagy.

Next, we explored the possible mechanisms underlying the AhR-regulated autophagy. We have previously reported that AhR modulates allergen-induced epithelial TGF-β1 secretion and activation (29, 45). This finding led us to hypothesize that TGF-β1 may be a major player in AhR-mediated autophagy. Indeed, we found increased levels of TGF-β1 in supernatants of cockroach allergen–treated HBECs, which were further enhanced in HBECs with AhR knockdown. Furthermore, we investigated whether the AhR-regulated TGF-β1 can directly induce autophagy. As expected, HBECs showed an increased autophagy after exposed to different doses of recombinant TGF-β1. In contrast, we found that cockroach allergen–induced autophagy was almost completely blocked when TGF-β1 was deleted by using TGF-β1 neutralizing antibody (α-TGF-β1). These studies suggest that TGF-β1 is required in AhR-regulated cockroach allergen–induced autophagy.

In addition to TGF-β1, we expanded our studies to identify novel genes and pathways that are involved in AhR-mediated autophagy and airway inflammation. We analyzed the transcriptional profiles of AT2 cells isolated from the lungs of *AhR^f/f^* and *Sftpc-cre;AhR^f/f^* mice. Although there were different comparisons between groups, the major comparison was between CRE-treated AT2 cells derived from *Sftpc-cre;AhR^f/f^* mice and *AhR^f/f^* mice with a total of 717 differentially expressed mRNAs identified. Of these, both AhR downstream genes Ahrr and Cyp1b1 showed significant reduction in AT2 cells of *Sftpc-cre;AhR^f/f^* mice, supporting the AhR deficiency in AT2 cells and proper experimental approaches we used in this study. In addition, we found our previously identified genes *COX-2* (47) and *Slc26a4* (38) that are associated with allergic airway inflammation. Furthermore, GO functional enrichment analysis based on these DEGs identified autophagy as one of the most enriched GO terms. Among these autophagy-related genes, *Calcoco2*, also named as *NDP52* (nuclear dot protein 52), is an essential selective autophagy adaptor that initiates selective autophagy through recruiting ULK and TBK1 kinase complexes (48). Recruitment of *Calcoco2* has also been suggested to amplify PTEN Induced Kinase 1 (PINK1)/Parkin mitophagy signaling (49). Our study demonstrated that *Calcoco2* was highly expressed in the lung tissues and AT2 cells of our asthma mouse model as determined by immunofluorescence staining. In contrast to *Calcoco2*, *S100a9* is downregulated in AT2 cells of *Sftpc-cre;AhR^f/f^* mice compared with *AhR^f/f^* mice. S100a9 is known as damage-associated molecular pattern, which can be induced in many cells under inflammatory condition (76). Interestingly, studies have highlighted a role for S100a9 in inducing pro-inflammatory cytokine release and in the ROS-dependent activation of the NLRP3 inflammasome (76). However, it remains unclear whether S100a9 is also involved in the allergen-induced and ROS-dependent activation of autophagy. In addition, we have also identified several other significant GO terms, including ubiquitin-protein transferase activity, cell activation, and cilium-related terms (e.g., movement, assembly, organization, and cilium). Those identified biological processes are clearly worth to be investigated in the future for their contributions to the AhR-mediated airway inflammation.

**Figure 9:**
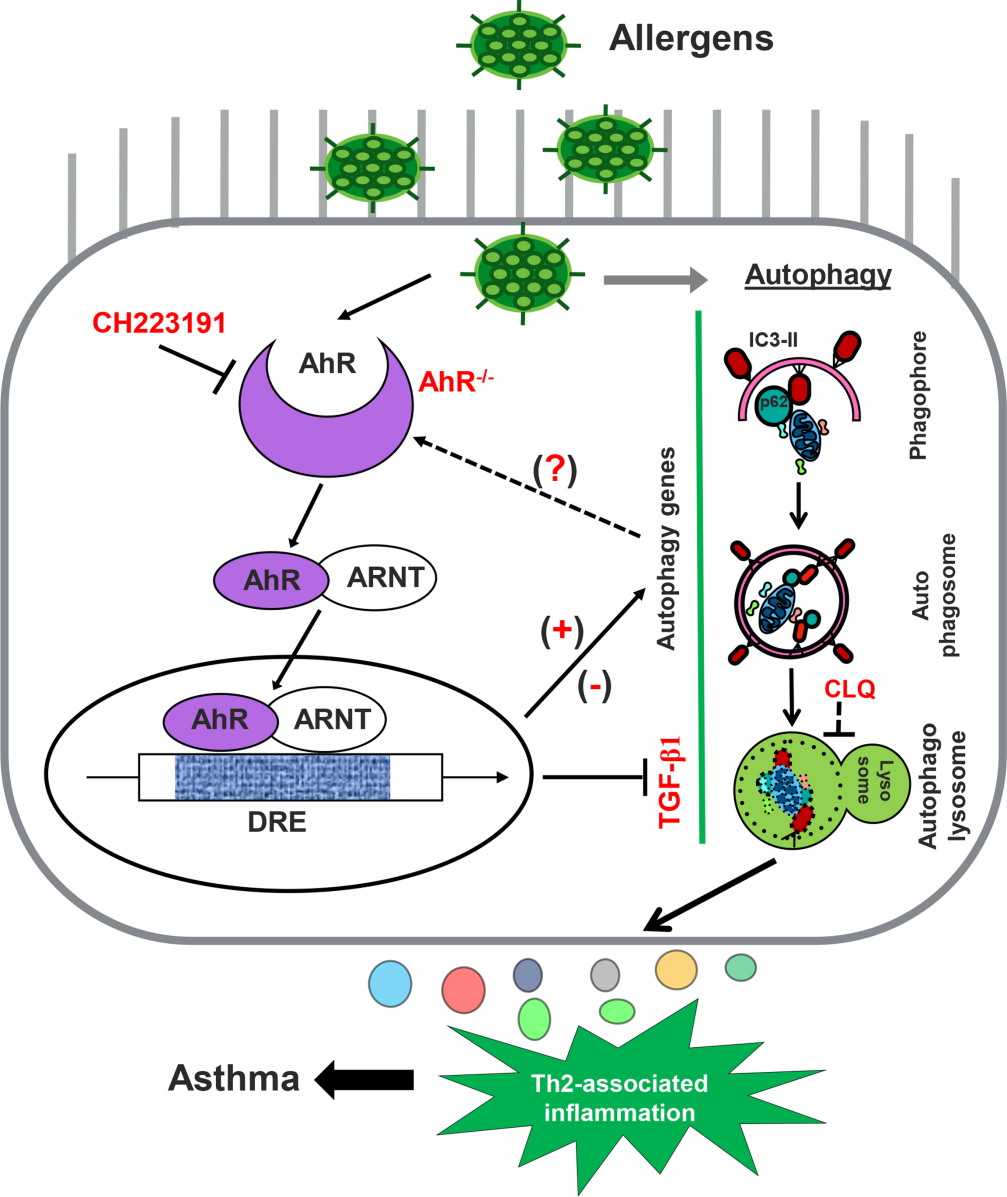
Graphic summary. AhR in AT2 cells regulates cockroach allergen–induced autophagy. Cockroach allergen can induce autophagy that is critical in controlling proinflammatory mediator release, Th2-associated airway inflammation, and, subsequently, allergic asthma. AhR in AT2 cells inhibits cockroach allergen–induced autophagy through either controlling TGF-β1 release or AhR-regulated autophagy-associated genes [either upregulated (e.g., *CALCOCO2*) or downregulated (e.g., *S100a9*)].

Taken together, we used our newly generated *Sftpc-Cre;AhR^f/f^* mice and provided a strong evidence that AhR in AT2 cells protects against cockroach allergen–induced airway hyperresponsiveness and Th2-associated airway inflammation. Further *in vivo* and *in vitro* studies suggest that cockroach allergen can induce autophagy in AT2 cells, and AhR in AT2 cells can prevent cockroach allergen–induced autophagy. Most intriguingly, autophagy inhibition attenuated cockroach allergen–induced airway inflammation and Th2-associated cytokines. Mechanistically, we identified a functional axis of AhR-TGF-β1 that is critical in driving allergen-induced cellular autophagy. Furthermore, our GO functional enrichment analysis on these DEGs from the RNA-seq analysis suggested that autophagy is one of the most enriched GO terms, and the autophagy adaptor *CALCOCO2* and *S100a9* are the major AhR-regulated autophagy genes in cockroach allergen–treated AT2 cells (**Figure 9**, graphic summary). However, the regulation of AhR on these identified autophagy genes and the exact role of these genes in the pathogenesis of asthma remain unclear, thus warranting further, in-depth investigations. One of the major limitations is the lack of primary human AT2 cells. Instead, we used HBECs, an immortalized cell line under submerged cultures, for *in vitro* studies, which may not truly reflect the real changes seen in AT2 cells. However, our findings in AT2 cells may also apply to the bronchial epithelial cells. Collectively, these findings highlight a critical role for AhR in AT2 cells in preventing allergic airway inflammation through regulating cellular autophagy.

## Data availability statement

The datasets presented in this study can be found in online repositories. The name of the repository and accession number can be found below: National Center for Biotechnology Information (NCBI) Gene Expression Omnibus (GEO), https://www.ncbi.nlm.nih.gov/geo/, GSE205818.

## Ethics statement

The animal study was reviewed and approved by The Johns Hopkins University Animal Care and Use Committee.

## Author contributions

JW, YLZ, XZ, WT, RW, YS, and YZ performed experiments, analyzed data, and review the manuscript. PG designed and supervised the study, and wrote the manuscript. All authors read and approved the final version of the manuscript.

## Funding

This work was supported by grants from the US National Institutes of Health (NIH) (1R01AI153331 and R01AI141642 to PG). This study was also supported by grants from the National Natural Science Foundation of China (31900667 to WT) and Shenzhen Scientific Technology Basic Research Project (JCYJ20190812171617278 to WT).

## Conflict of interest

The authors declare that the research was conducted in the absence of any commercial or financial relationships that could be construed as a potential conflict of interest.

## Publisher’s note

All claims expressed in this article are solely those of the authors and do not necessarily represent those of their affiliated organizations, or those of the publisher, the editors and the reviewers. Any product that may be evaluated in this article, or claim that may be made by its manufacturer, is not guaranteed or endorsed by the publisher.

## Abbreviations

CRE, cockroach extract; AhR, aryl hydrocarbon receptor; WT, wild-type; BALF, bronchoalveolar lavage fluid; PAS, Alcian Blue periodic acid–Schiff; RT-PCR, quantitative real-time PCR; AHR, airway hyperresponsiveness; BSA, bovine serum albumin; FBS, fetal bovine serum; H&E, hematoxylin and eosin; AT2, type II alveolar epithelial cells; HBEC, human bronchial epithelial cell; CLQ, chloroquine; GO, Gene Ontology; NLRP3, nucleotide-binding oligomerization domain (NOD)–like receptor family pyrin domain-containing 3.
