# Supplementary material for: Type II alveolar epithelial cell aryl hydrocarbon receptor protects against allergic airway inflammation through controlling cell autophagy
Source: Front Immunol. 2022 Jul 22;13:964575. doi: 10.3389/fimmu.2022.964575 (PMC9355649; doi:10.3389/fimmu.2022.964575)
Supplement: Supplementary file 1 [file DataSheet_1.pdf]

Table S1. Antibodies used for western blot (WB) and immunofluorescence (IF) staining

Target	Species	Clone	Assay (dilution)	Company
Atg5	mouse	603813	IF (1:100)	R&D systems
Beclin1	mouse	G-11	IF (1:50)	Santa Cruz Biotechnology
LC3B	Rabbit	1251B	IF (1:100)	R&D systems
P62	Rabbit	NBP1-48320	IF (1:100)	NOVUSBIO
Sftpc	mouse	4A10	IF (1:200)	Abnova
Sftpc	Rabbit	AB3786	IF (1:200)	Millipore
S100A9	Goat	AF2065-SP	IF (1:100)	R&D systems
Calcoco2	Mouse	F-6	IF (1:50)	Santa Cruz Biotechnology
AhR	Rabbit	GTX129013	IF (1:100)<br>WB (1:1000)	GeneTex
β-Actin	Mouse	2F1-1	WB (1:1000)	BioLegend

Table S2. primers for RT-PCR

Gene	Species	NCBI GeneID	Sequence (5'-3')
ATG5	Mus	11793	Fwd: GACAAAGATGTGCTTCGAGATGTG
			Rev: GTAGCTCAGATGCTCGCTCAG
GAPDH	Mus	14433	Fwd: GGAGCGAGATCCCTCCAAAAT
			Rev: GGCTGTTGTCATACTTCTCATGG
LC3A	Mus	66734	Fwd: AGCTTCGCCGACCGCTGTAAG
			Rev: CTTCTCCTGTTCATAGATGTCAGC
LC3B	Mus	67443	Fwd: CGGAGCTTTGAACAAAGAGTG
			Rev: TCTCTCACTCTCGTACACTTC
Beclin1	Mus	56208	Fwd: CTGAAACTGGACACGAGCTTCAAG
			Rev: CCAGAACAGTATAACGGCAACTCC
p62	Mus	18412	Fwd: TGTGGAACATGGAGGGAAGAG
			Rev: TGTGCCTGTGCTGGAACTTTC
AhR	Mus	11622	Fwd: ATGAGCAGCGGCGCCAACAT
			Rev: AGGCTGGCCAGGCGGTCTAA
Sh3bp4	Mus	98402	Fwd: AAGTCGGAGGGGACTCTGATA
			Rev: CGTGGGGTTGTCTACGAGC
Calcoco2	Mus	76815	Fwd: GCCCCATACCTACCTTGCTG
			Rev: TCGAGGGATGAACTTTTCAGTG
Prkd1	Mus	18760	Fwd: GGGGGCATCTCGTTCCATC
			Rev: GTGCCGAAAAAGCAGGATCTT
Usp10	Mus	22224	Fwd: AACCCACAGTATATCTTTGGCG
			Rev: CCCTCACTAGGTTCGATGACTTC
S100a9	Mus	20202	Fwd: ATACTCTAGGAAGGAAGGACACC
			Rev: TCCATGATGTCATTTATGAGGGC
AhR	Hemo	196	Fwd: ACATCACCTACGCCAGTCGC
			Rev: TCTATGCCGCTTGGAAGGAT
Sh3bp4	Hemo	23677	Fwd: ACCCTGATTGACCTGAGCGA
			Rev: GGGGTTGTCTACGAGCAAGG
Calcoco2	Hemo	10241	Fwd: TGAAGGAGGCGCAAGACAAAA
			Rev: CATCTGCTGTTGCTCCAAGGT
Prkd1	Hemo	5587	Fwd: CTTTTTCGCCATGACCCTACC
			Rev: GGAAGCTGACAAGACCACTTCA

Usp10	Hemo	9100	Fwd: ATTGAGTTTGGTGTCGATGAAGT
			Rev: GGAGCCATAGCTTGCTTCTTTAG
S100a9	Hemo	6280	Fwd: CTGGACACAAATGCAGACAAG
			Rev: GTCACCCTCGTGCATCTT
β-Actin	Hemo	60	Fwd: AGAAAATCTGGCACCACACC
			Rev: CAGAGGCGTACAGGGATAGC
ATG5	Hemo	9474	Fwd: AACTGAAAGGGAAGCAGAACCA
			Rev: CCATTTCAGTGGTGTGCCTTC
LC3A	Hemo	84557	Fwd: CCAGCAAAATCCCGGTGAT
			Rev: TGGTCCGGGACCAAAAACT
LC3B	Hemo	81631	Fwd: ACCATGCCGTCGGAGAAG
			Rev: GGTTGGATGCTGCTCTCGAA
Beclin1	Hemo	8678	Fwd: GGCTGAGAGACTGGATCAGG
			Rev: CTGCGTCTGGGCATAACG
p62	Hemo	8878	Fwd: GGTACCCGCCACCATGGCGTCGCTCACCGTGAA
			Rev: TCTAGACAACGGCGGGGGATGCTTTG
